# A Study on the Effectiveness of Deep Learning-Based Anomaly Detection Methods for Breast Ultrasonography

**DOI:** 10.3390/s23052864

**Published:** 2023-03-06

**Authors:** Changhee Yun, Bomi Eom, Sungjun Park, Chanho Kim, Dohwan Kim, Farah Jabeen, Won Hwa Kim, Hye Jung Kim, Jaeil Kim

**Affiliations:** 1 National Information Society Agency, Daegu 41068, Republic of Korea; 2 School of Computer Science and Engineering, Kyungpook National University, Daegu 41566, Republic of Korea; 3 Department of Artificial Intelligence, Kyungpook National University, Daegu 41566, Republic of Korea; 4 Department of Radiology, Kyungpook National University Chilgok Hospital, Kyungpook National University, Daegu 41404, Republic of Korea

**Keywords:** breast cancer, ultrasonography, deep learning, anomaly detection, autoencoder

## Abstract

In the medical field, it is delicate to anticipate good performance in using deep learning due to the lack of large-scale training data and class imbalance. In particular, ultrasound, which is a key breast cancer diagnosis method, is delicate to diagnose accurately as the quality and interpretation of images can vary depending on the operator’s experience and proficiency. Therefore, computer-aided diagnosis technology can facilitate diagnosis by visualizing abnormal information such as tumors and masses in ultrasound images. In this study, we implemented deep learning-based anomaly detection methods for breast ultrasound images and validated their effectiveness in detecting abnormal regions. Herein, we specifically compared the sliced-Wasserstein autoencoder with two representative unsupervised learning models autoencoder and variational autoencoder. The anomalous region detection performance is estimated with the normal region labels. Our experimental results showed that the sliced-Wasserstein autoencoder model outperformed the anomaly detection performance of others. However, anomaly detection using the reconstruction-based approach may not be effective because of the occurrence of numerous false-positive values. In the following studies, reducing these false positives becomes an important challenge.

## 1. Introduction

Recently, deep learning (DL), a branch of machine learning, has attracted considerable attention. This is a technology for hierarchically learning numerous data features through a deep artificial neural network(ANN), extracting from simple features of input data to complex features [1]. In addition, DL performs well in analyzing various data types, such as video, voice, and text. Moreover, it can be applied to various areas, such as image classification, object detection, language translation, sentence classification, voice automatic generation and composition, robotics, medical image analysis, and cybersecurity [2].

In the medical field, various medical imaging techniques, such as magnetic resonance imaging (MRI), X-ray, computed tomography, ultrasound, and endoscopy are used for numerous complicated medical imaging analyses because of their improved diagnosis rates and reduced screening time based on the consistency, scalability, and accuracy of DL. However, it is challenging to apply DL models to numerous medical images using various types of medical equipment without additional information from experts. Consequently, a method for self-learning the inherent features from numerous images without additional expert opinion and maximizing discrimination via a minimal amount of expert judgment has been developed recently [3].

Among the above medical imaging techniques, ultrasound is one of the key diagnostic imaging techniques for the physical examination of various organs, such as abdominal organs, breasts, musculoskeletal systems, heart, and blood vessels [3]. Furthermore, ultrasonic waves can be imaged in real-time and used with existing resources without building a separate environment. However, the quality and interpretation of an image may differ depending on the operator [3,4] and the false-positive rate (FPR), which is the probability of judging a disease-free normal region as an anomaly with a high value [5]. In particular, in breast ultrasonography, it is difficult to detect lesions and accurately diagnose them with a false-negative rate of 50% in dense breasts with a large quantity of mammary tissue and a fairly small quantity of fat [5]. To overcome these limitations, DL technology has been employed to effectively extract biometric information or elaborately visualize anomaly information of organs similar to masses and tumors to aid diagnosis.

Therefore, in this study, DL models were applied to breast ultrasound images to learn the image features. Using anomalous data, the results of applying deep learning-based anomaly detection methods for ultrasound images were verified. Thus, DL-based anomalous region detection technology can automatically detect anomalous regions with tumors or masses in ultrasound images. Moreover, we aim to study the effectiveness of this technology in practical applications, e.g., whether it can be used as a computer-aided diagnostic tool to detect anomalous regions more quickly in ultrasound diagnosis and more accurately by visually presenting the anomalous region to the user than those of the other tools.

## 2. Related Work

### 2.1. Deep Learning-Based Anomaly Detection

An anomaly is generally defined as the contrary conception of the normal defined in a field or problem. Anomalies can be largely categorized into point, contextual, and collective anomalies [5]. Point anomalies represent irregularities or diversions; individual data can be linked from given data without a particular interpretation and are considered anomalies. Contextual anomalies are also called conditional anomalies; data are judged to be anomalous in certain situations and are identified in consideration of contextual, behavioral, and operational attributes. Collective anomalies may not be anomalies for individual data; however, data related to each other show anomalous characteristics within an entire group and are judged as anomalous.

Anomaly detection means finding an unusual pattern unless the expected behavior in the data is followed, defining a region representing normal behavior, and considering data that do not belong to the specific region as anomalous and finding them [6]. These detection methods have long been applied in various fields, e.g., medicine, transportation, cyber intrusion, telephone or insurance fraud, and industrial control system detection, playing a crucial role as the demand increases and applications become widespread [7].

DL is a type of ANN that resembles human cognitive function as a machine learning technique [8]. This is to achieve flexibility by learning how to express data in an overspread hierarchical structure and ensure excellent performance in learning complex data characteristics such as high-dimensional, temporal, spatial, and graphic data on its purpose of analysis [7]. DL-based anomaly detection applies DL technology to the anomaly detection method. A deep ANN algorithm comprising artificial neurons stacked between the input and output layers is applied to determine whether there is an anomaly.

This method is further classified into supervised, semisupervised, and unsupervised learning according to the learning approach. Besides, this method is utilized to supervise outlier detection according to the presence or absence of label data, which is used for learning data [6].

#### Unsupervised Deep Anomaly Detection

Supervised and semisupervised deep anomaly detection approaches require securing labels for learning data. Because obtaining labeled data is complex, research is actively being conducted to enable learning without obtaining separate label data, assuming that most data are normal [9]. The objective of unsupervised anomaly detection is to detect previously unseen rare objects or events without prior knowledge about them, meaning it only requires a single labeling process to train a model. Consequently, high accuracy is not achieved because the restoring performance of the original data depends on the degree of compression of input data.

**Figure 1:**
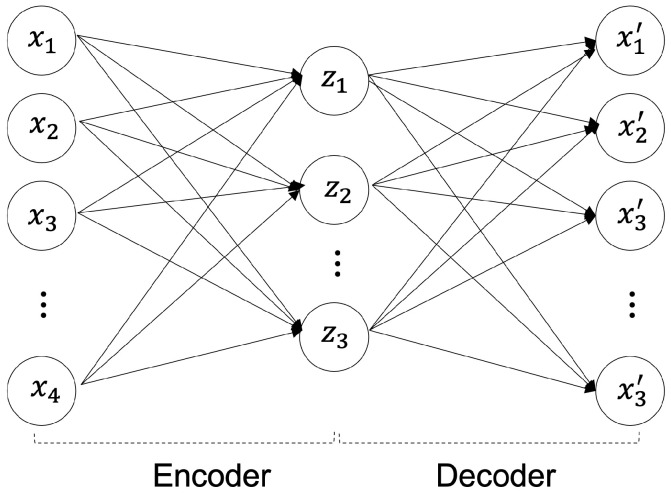
Autoencoder (AE) Architecture.

The reconstruction methodology for deep anomaly detection has been implemented for unsupervised-based deep anomaly detection. The authors of [10] assumed that learned traditional structures are well-remodeled and reconstructed; however, abnormal structures were difficult to reconstruct. Specifically, in images, a significant difference was visible between the input data and the anomalous region reconstructed using the data that can be determined using an object. The core model of unsupervised-based deep anomaly detection is an AE [11]. As shown in Figure 1, an AE is a generative unsupervised DL algorithm for reconstructing high-dimensional input data. An AE uses an NN with a narrow bottleneck layer in the middle that contains the latent that compresses features and then decodes data to reconstruct the original input. The encoder maps the input data features to a low-dimensional latent space, and the decoder is trained to restore the low-dimensional features most similar to the input data through reverse processing.

The encoder maps high-dimensional data into a low-dimensional latent space as shown in Equation (1), and the decoder reconstructs and restores the compressed low-dimensional data as shown in Equation (2) into high-dimensional data [1]. In Equations (1) and (2), the encoder parameters are {W,b} and the decoder parameters are $\left\{W^{\prime },b^{\prime }\right\}$. The activation function is α [1].

(1) z=encoder(x)=α(Wx+b)

(2) $$x^{\prime }=decoder\left(z\right)=\alpha \left(W^{\prime }z+b^{\prime }\right)$$

As shown in Equation (3), the purpose of the AE model is to minimize the reconstruction errors using the difference between the restored images and the input image that mainly uses mean square error (MSE) and cross-entropy error.

(3) $$L\left(x,x^{\prime }\right)=argmin\frac{1}{n}\sum_{i=1}^{n}{∥x-x^{\prime }∥}^{2}$$

We consider three typical AE models applied to unsupervised-based deep anomaly detection: variational AE (VAE), general adversarial network (GAN), and sliced-Wasserstein AE (SWAE).

**Figure 2:**
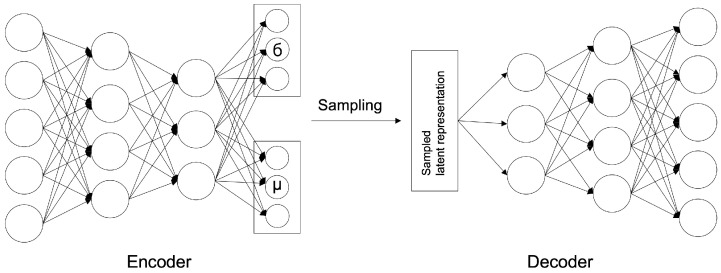
VAE Architecture.

The VAE model was proposed by D. Kingma and M. Welling [12] in 2014; it is a generative model that learns the probability distribution of data and generates new data from the learned probability distribution. The structure is shown in Figure 2 and comprises a network structure of an encoder and a decoder, as shown in the AE model. The encoder extracts potential features by abstracting input data, and the decoder restores these potential features to the original data. At this time, the decoder generates data on the premise of a normal distribution with the average (μ) and variance (σ) of the latent features created by the encoder as parameters.

The loss function of the VAE model is shown in Equation (4), which computes the errors in the two optimization tasks. It comprises a sum of reconstruction errors, indicating how well the input image has been restored, and Kullback–Leibler divergence (KLD) errors, indicating how closely the latent variable matched the Gaussian distribution, i.e., the latent space probability distribution.

(4) $$L_{i}\left(\theta ,\phi \right)=-E_{z}∼q_{\theta }\left(z|x_{i}\right)\left[\log_{p_{\phi }}\left(x_{i}|z\right)\right]+KL\left(q_{\theta }\left(z|x_{i}\right)|p\left(z\right)\right),$$

where *x* is an input value, and *z* represents a sampled latent variable. θ is the encoder parameter, ϕ is the decoder parameter; the encoder and decoder can be expressed as $q_{\theta }\left(z|x\right)$ and $p_{\phi }\left(x|z\right)$, respectively.

The SWAE model enables the shaping of the latent space distribution into a samplable probability distribution without the need to train an adversarial network [12]. Similar to the VAE model, the sample data distribution is enforced. However, in the normalization process, there is a difference between the usage of the Wasserstein distance (WD) and not the KLD. Both the KLD and WD measure the distance between probability distributions. However, the KLD is θ when the two probability distributions overlap, as shown in Equation (5), and +∞ when they do not overlap. Thus, learning becomes problematic if the probability distribution is not continuous. However, the WD (EM distance) maintains a constant $\left|\theta \right|$ regardless of whether the two probability distributions overlap, as shown in Equation (6). Hence, it is easy to use it in learning because probability distributions that do not converge with other distances or divergences can converge with it.

(5) $$KL\left(P_{\theta }∥P_{0}\right)=KL\left(P_{0}∥P_{\theta }\right)=\left\{\begin{array}{cc} +\infty & \mathrm{if}\;\theta \neq 0,\\ 0 & \mathrm{if}\;\theta =0 \end{array}\right.$$

(6) $$W(P_{0},P_{\theta })=|\theta |$$

To minimize the sliced-WD (SWD) between the distribution of encoded learning data and the prior distribution, the distance used in sliced-Wasserstein is the same as that in Equation (7). It refers to the lower limit when the expected value of the distance is the smallest in the combined probability distributions $\gamma \left(x,y\right)$ combining the two probability densities $P_{r}$ and $P_{g}$.

(7) $$W\left(P_{r},P_{g}\right)=\underset{\gamma \in \Pi (P_{r},P_{g})}{\inf}\left(E_{(x,y)\gamma }{\left[{∥x-y∥}^{p}\right]}^{\frac{1}{p}}\right)$$

However, because it is impossible to find the minimum in all combinations of probability distributions, we calculate the value for the 1-Lipschitz function ${∥f∥}_{L}\leq 1$, which is the upper limit where the average rate of change between any two points does not exceed 1, using the Kantorovich–Rubinstein equation:

(8) $$W\left(P_{r},P_{g}\right)=\underset{{∥f∥}_{L}\leq 1}{\sup}E_{x∼P_{r}}\left[f\left(x\right)\right]-E_{x∼P_{g}}\left[f\left(x\right)\right]$$

The SWD projects high-dimensional probability densities such as $P_{r}\;and\;P_{g}$ in the distribution of Equation (8) from the WD into one-dimensional (1D) peripheral distributions and compares these peripheral distributions through the WD.

For the two probability distributions *R* and *G*, the Wasserstein-2 distance is calculated as Equation (9), and the SWD is approximated to $W_{2}^{2}$ as shown in Equation (10) and optimized as Equation (11).

(9) $$W_{2}^{2}\left(R,G\right)=\frac{1}{\left|G\right|}\min_{M}\sum_{i=1}^{\left|G\right|}\sum_{j=1}^{\left|R\right|}M_{i,j}{∥R_{j}-G_{i}∥}_{2}^{2},M:\int ,doubly\;stochastic$$

(10) $$\tilde{W_{2}^{2}}\left(R,G\right)=\int_{w\in \Omega }W_{2}^{2}\left(R^{w},G^{w}\right)dw,R^{w}=w^{T}R_{i_{i=1}}^{\left|R\right|},G^{w}=w^{T}G_{i_{i=1}}^{\left|G\right|},\Omega :unit\;sphere$$

(11) $$\min_{\theta }\frac{1}{\hat{\left|\Omega \right|}}W_{2}^{2}\left(R^{w},G^{w}\left(\theta \right)\right)dw$$

The 1D peripheral distribution of the high-dimensional probability densities may be defined as follows:

(12) $$R_{PX}\left(t;\theta \right)=\int_{X}PX\left(x\right)\delta \left(t-\theta •x\right)dx,\forall \theta \in S^{d-1},\forall t\in R,$$

where $S^{d-1}$ means a unit sphere of d-dimensional, and for fixed $\theta \in S^{d-1}$, $R_{PX}\left(•;\theta \right)$ is a 1D slice of PX distribution. That is, $R_{PX}\left(•;\theta \right)$ is obtained by integrating a hyperplane PX orthogonal to θ. The following Equation (13) is the sliced-WD defined from the peripheral distribution of Equation (12).

(13) $$SW_{c}\left(PX,PY\right)=\int_{S^{d-1}}W_{c}\left(R_{PX}\left(•;\theta \right),R_{PY}\left(•;\theta \right)\right)d\theta$$

**Figure 3:**
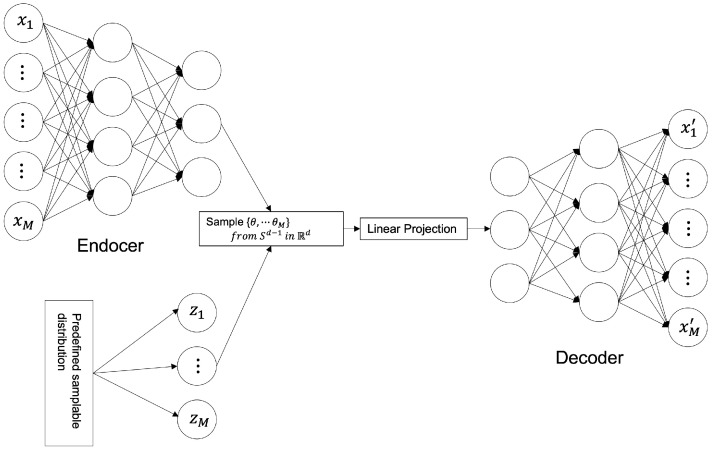
SWAE Architecture.

According to Soheil Kolouri [13], the SWAE is calculated as follows to optimize the model to the minimum SWD value:

(14) $$argmin_{\phi ,\psi }W_{c}\left(PX,PY\right)+\lambda SW_{c}\left(pz,qz\right),$$

where ϕ represents an encoder, ψ represents a decoder, PX represents a data distribution, PY represents a distribution of data through an encoder and a decoder; pz is the encoded data distribution, and qz represents a predefined sampling distribution; λ represents the relative importance of the loss function. The model structure is shown in Figure 3.

Most DL-based anomaly detection models learn using one of the aforementioned three learning approaches and determine whether it is abnormal through output values. According to the result, an abnormal score that can be determined based on a specific reference value is defined for a given problem to determine its abnormality.

### 2.2. Deep Learning-Based Anomaly Detection for Medical Images

In the medical field, DL-based anomaly detection methods have been applied to improve classification performance by learning the characteristics of complex and abstract medical images and spatially transforming lesions to contribute to the characteristics, which is helpful for prevention treatments [14].

Data imbalance due to the variety of data is a common issue in the medical field. It is challenging to collect disease data compared with normal data due to practical limitations in detecting and classifying lesions. Recently, DL methods have been implemented for anomaly detection for various medical images modalities, such as brain MRI, retinal optical coherence tomography (OCT), hand X-ray, chest X-ray, skin disease, and muscle ultrasound [15,16,17,18,19,20,21,22,23,24,25,26].

Unsupervised anomaly detection based on implicit field learning was recently proposed for high-resolution three-dimensional volume images [27]. The implicit field learning was implemented to learn a mapping of latent features and coordinates to a data point intensity class so that the encoding module preserves as much information as possible in the original image. The implicit field learning approach with AE achieved state-of-the-art performance in anomaly detection for brain cancer MRI. GAN-based architectures have also been employed in various anomaly detection studies. In [28], the GANomaly architecture was applied to detect chronic brain infarcts. In [29], a unified GAN and VAE architecture was proposed to identify chest radiographs with abnormal lesions.

DL methods, especially AE and GAN architectures, learn normal image patterns of human organs in medical images without lesions. In the process of reconstructing a given image, they have the advantage of using the difference between the input image and the reconstructed image to determine the abnormality of the input. However, although various AEs have been proposed, the FPR is still high in pixel-wise anomaly detection. In this study, the effectiveness of the SWAE in anomaly detection, which is known to have better reconstruction quality than other AE variants, is validated through comparative studies with the VAE and conventional AE models.

## 3. Materials & Methods

### 3.1. Materials

In this study, we retrospectively collected 1147 breast ultrasound images comprising 947 normal breast ultrasound images and 200 abnormal ultrasound images from Kyungpook National University Hospital in the Republic of Korea. The images consist of 113 benign tumors and 87 malignant tumors. The size of all data is 224×224×3; 853 normal breast ultrasound data and 94 normal data for model training and verification. Data with anomalous region (region of interest: ROI) label values were used for model evaluation.

The ultrasound images used in the experiment were cut into specific areas. Some normal ultrasound images were used for learning via applying Gaussian filters for noise removal, and gamma correction with 0.5 and 1.5 gamma values, which decide to express the dark areas of ultrasound in more detail. The input data were used by dividing the values of 0–255 pixels into 255 values and converting them into values between 0 and 1.

### 3.2. Reconstruction-Based Anomaly Detection

**Figure 4:**
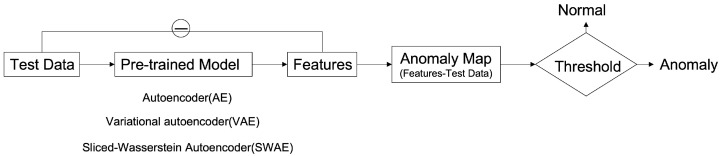
Deep Learning-based Anomalous Region Detection Process.

The method of detecting an anomalous region applied in this study is to detect an unrestored region by considering it as abnormal using an error image between an input image and a reconstructed image (Figure 4). The learning process uses a modified SWAE model based on AE, a representative generation model of ANNs, and the conventional AE, which obtains latent features for the summit through input. In the evaluation process, anomalous data are input to the learned model, and an anomalous region is detected through the restored results. The difference between the input image and the restored image is calculated to derive an anomaly map, which is an error image. The anomaly map is binary divided based on a specific threshold to detect the anomalous region. This process was applied to the three models to compare and analyze their detection performances and investigate the factors influencing anomalous region detection in breast ultrasound images.

#### 3.2.1. Hyperparameter Tuning

In this study, the hyperparameters of the implemented models are as shown in Table 1, Table 2 and Table 3. We tuned the hyperparameters by the grid search method.

#### 3.2.2. Model Architecture of Anomaly Detection Model for Breast Ultrasound

**Figure 5:**
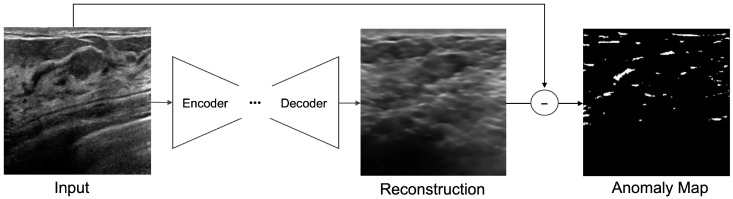
Anomaly detection by pixel difference between an original image and reconstructed image on ultrasonography.

The implemented models comprise encoders and decoders with multiple hidden layers. In the learning process, the encoders map normal ultrasound images into low-dimensional spaces to represent them as key features of the latent space; meanwhile, the decoders update and restore weight to some extent according to input. The process for detecting the anomalous region calculates a pixel unit error over the reconstructed, restored image and the input image (Figure 5). The anomaly map detects an anomalous region by binary division based on a specific threshold. It considers the region abnormal if it is larger than the threshold value and normal otherwise.

##### Autoencoder (AE) Model

**Figure 6:**
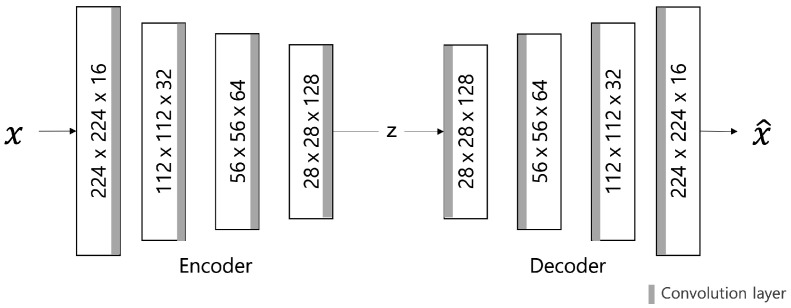
AE model architecture.

Figure 6 describes the AE model, comprising different filter sizes and convolutional layers that are added to the encoder and decoder to extract features. Therefore, the batch normalization layer is used to normalize the power value. The LeakyRelu activation function is used with a slight slope to convert the calculated input value into the power value. In this model, input data are converted to values between 0 and 1 through normalization, and a sigmoid function is used as the output layer.

The loss value *L* of the AE model is calculated using the L1 distance loss function to indicate the abnormal score by the difference in pixel values. This is calculated as the sum of the absolute values of the difference between the restored image $\hat{x}$ and the input image *x* (Equation (15); the smaller the loss value, the better the model performance. The Adam optimizer is used for model optimization. The learning rate is set to the maximum initial value of 0.0002. The cosine annealing method, which can improve accuracy by adjusting the learning rate in a cosine function, is applied.

(15) $$L\left(x,\hat{x}\right)=\sum_{i=1}^{n}\left|x_{i}-\hat{x_{i}}\right|$$

##### Variational Autoencoder (VAE) Model

**Figure 7:**
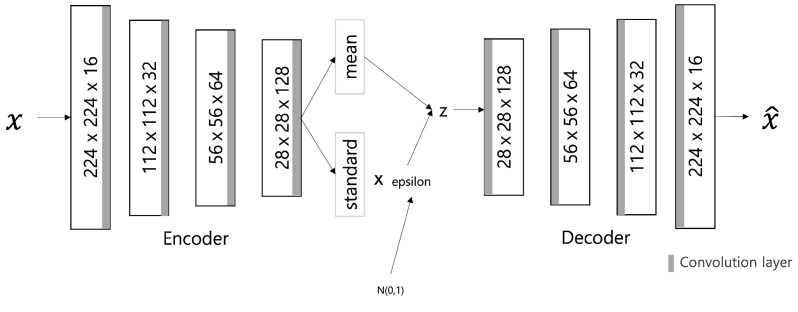
VAE model architecture.

The VAE model comprises an encoder and a decoder similar to the AE model. The only difference is the AE model is used to map Gaussian distribution and noise for normalization to the latent space (Figure 7). It is to generate similar data using the latent variable *z* by allowing the encoder to return the distribution of the latent space instead of a single point. Changing the parameter can be ideal for the probability distribution. In this case, the distribution returned from the encoder is close enough to the standard normal distribution. In this study, we assumed a Gaussian distribution. Because the immediate differential calculation is impossible in the latent variable sampling stage. Thus, the latent variable is converted into z=μ+ϵσ(sampleϵ∼N(0,1)) using the reparameterization trick for optimization to enable backpropagation.

The input data are converted into values between 0 and 1 through normalization, and the output layer of the model uses a sigmoid function. The loss value *L* for model optimization comprises the sum of reconstruction errors using L1 distances as shown in Equation (16) and the KLD terms for normalization. As in the AE model, the learning rate is set to 0.0002 and adjusted by applying cosine annealing for accurate learning. The parameters are updated using the Adam optimizer for model optimization.

(16) $$\begin{array}{cc} L & =Regularization\;Parameter+Reconstruction\;Error\\ \; & =D_{KL}\left(q_{\emptyset }\left(z|x\right)∥p_{\theta }\left(z|x\right)\right)+L\left(\theta ,\emptyset ,x\right)\\ \; & =D_{KL}\left(N\left(\mu ,\sum \right)∥N\left(0,1\right)\right)+E_{q_{\emptyset }}\left[\log_{p_{\theta }}\left(x|z\right)\right]\\ \; & =-\frac{1}{2}\sum_{j=1}^{J}\left(1+\log\left(\sigma_{j}^{2}\right)-\mu_{j}^{2}+\sigma_{j}^{2}\right)+E[\sum_{i=1}^{D}\left(x_{i}\log_{y_{i}}+\left(1-x_{i}\right)•\log\left(1-y_{i}\right)\right] \end{array}$$

##### SWAE Model

**Figure 8:**
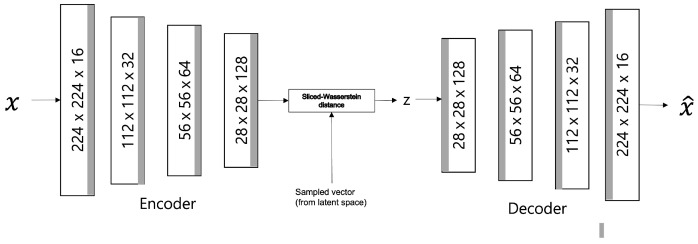
SWAE model architecture.

Similar to the VAE model, the SWAE model is a generative model comprising an encoder and a decoder, which allows the latent space to be formed into a sampling probability distribution. However, the only difference is normalizing reconstruction losses using the SWD between the encoded learning sample distribution and the predefined sampling distribution. Figure 8 shows the SWAE architecture.

Ultrasonography data converted to values between 0 and 1 are used as input, and the configuration and output of each model layer are configured the same as those of the AE and VAE models. The loss value *L* is calculated as the sum of the reconstruction error and the SWD of the 1D projection for normalization (Equation (17)). The maximum value of the learning rate is set to 0.0002, and cosine annealing is applied and adjusted to increase accuracy.

(17) $$\begin{array}{cc} L & =L_{rec}+Sliced-Wasserstein\;distance\\ \; & =\frac{1}{n}\sum_{i=1}^{n}{\left(x-\hat{x}\right)}^{2}+SW\left(P_{x},P_{\hat{x}}\right)\\ \; & =argmin_{Enc,Dec}W\left(P_{x},P_{\hat{x}}\right)+\lambda SW\left(pz,qz\right) \end{array}$$

In the loss function calculation, $L_{rec}$ evaluates the error between the input and reconstructed images as a pixel-by-pixel MSE, and the SWE is applied by projecting the difference between the encoded data distribution pz and predefined sampling distribution qz in dimensions.

(18) $$MSE=\frac{1}{n}\sum_{i=1}^{n}{\left(x_{i}-\hat{x_{i}}\right)}^{2}$$

#### 3.2.3. Validation of Anomaly Detection Method for Breast Ultrasonography

Anomalous data are input to the learned model to detect the anomalous region of an ultrasound image, and the output is a difference image between the restored and input images. The anomalous region is detected by a binary division based on a specific threshold. For performance verification, the ROI label data, extracted from a tumor region of the breast ultrasound image, is used. Indicators such as similarity (Dice), sensitivity (true-positive rate (TPR)), and FPR are calculated using overlapping pixel value information in the anomalous region of the label data and the binary-split image obtained from the models. Further, these indicators are employed to compare and analyze the detection results of each model. In addition, factors influencing the anomalous region detection results in an ultrasound image are identified.

##### Performance Evaluation of Anomaly Detection

In this study, three models were used to detect anomalous regions using the error value between the input and reconstructed images. This should restore the normal ultrasound image input for learning, and the abnormal ultrasound image input for testing should restore the anomalous region close to normal. The role of restoration is essential for successful anomalous region detection by applying a reconstruction-based approach to ultrasound images. Accordingly, the restoration results for each model for normal and abnormal ultrasound images are compared and analyzed using the root MSE (RMSE) values that minimize the error between the input and reconstructed images (Equation (19)).

(19) $$RMSE=\sqrt{\frac{1}{n}\sum_{i=1}^{n}{\left(Reconstruction-Input\right)}^{2}}$$

Restoration performance by RMSE value-based model can be considered as a model with improved learning when learning with normal data, a high RMSE value when evaluated with anomalous data, and failure to restore results and can be attributed to a well-trained model for anomalous region detection.

In addition, three indicators, Dice, TPR, and FPR, belonging to the overlap-based evaluation index group, were used to evaluate anomaly detection performance. Dice is calculated from Equation (20) using true positive (TP), false positive (FP), false negative (FN), and true negative (TN), which are components of the diffusion matrix. It is an indicator that checks the similarity with the correct answer by directly comparing the division results of the two images. TPR is an indicator of sensitivity, and by predicting the actual anomalous region abnormal, the anomalous region detection results can be confirmed. Moreover, FPR is an indicator of the normal region classified above [30]. Performance is measured based on the indicator values for each model derived by inputting anomalous data into the model, which are evaluation data. Indicator values are also compared and analyzed to verify whether the reconstruction-based approach of unsupervised learning is suitable for anomaly detection in ultrasound images.

(20) $$Dice=\frac{2TP}{2TP+FN+FP},\;TPR=\frac{TP}{TP+FN},\;FPR=\frac{FP}{FP+TN}$$

##### Analysis of Factor Influencing Anomalous Region Detection

To measure the anomaly detection performance of the reconstruction-based approach, we analyzed the effects of threshold setting and model-specific latent variables on reconstruction [17] and tumor and mass size of ultrasound images on anomaly detection.

As for the threshold for determining the anomalous region, the difference between the mean values of the individual anomaly maps and the overall anomaly map of the validation data is calculated using 94 normal data points for validation, as shown in Algorithm 1, and the maximum value calculated by applying the Relu function is set as the reference threshold [31]. However, in this study, the Relu function applied to obtain the threshold value treats the negative value of the vector as 0. Hence, the threshold value becomes relatively large, resulting in a region that treats the abnormality as normal. Therefore, by supplementing this, three additional thresholds, 0.1, 0.2, and 0.3, which can more accurately detect anomalous regions in ultrasound images, were applied and compared.
**Algorithm 1:** Find threshold for anomaly detection**Input:** anomaly map of validation dataset**Output:** threshold1:Max_relu←02:Calculate an average of anomaly map3:**for** *v* **in** validation set **do**4:    relu_th←ReLU(v−average)5:    **if** Max_relu<max(relu_th) **then**6:        Max_relu←max(relu_th)7:    **end if**8:**end for**    **return** Max_relu

Other influencing factors include the latent variable dimension of the latent space. The results are analyzed by limiting the structure of latent features through whether the encoder that generates latent variables for each model reduces dimensions. A reconstructed image is derived by varying the latent space dimensions of the three models. Anomalous region detection was performed by setting the latent space to a low dimension. In addition, the encoder and anomalous region detection results were confirmed by setting the latent space to a high dimension. Furthermore, changes in indicators according to the ROI sizes, such as masses and tumors of abnormal images used in the evaluation process, were examined. We also confirmed that ROI affects anomalous region detection.

## 4. Experimental Results and Analysis

### 4.1. Experimental Overview and Environment

In our experiment, AE, VAE, and SWAE models were implemented by applying the reconstruction-based approach of unsupervised learning. The detection performance of each model was measured. In addition, the effect of anomaly detection application in ultrasound was confirmed by comparison based on the performance evaluation values for each model.

The experimental environment used is the programming language Python 3.6.9 version, DL framework Pytorch 1.6 version, CUDA 10.0 version for GPU operation, and cuDNN 7.6.5 version library. A model’s learning, evaluation, and outcome analysis are performed in an environment using Intel(R) Core(TM) i7-1065G7 CPU @ 1.30 GHz 1.50 GHz and GeForce GTX Titan Xp 440.100 versions.

### 4.2. Evaluation of Anomalous Region Detection in Ultrasonography

#### 4.2.1. Reconstruction Performance by Model

**Figure 9:**
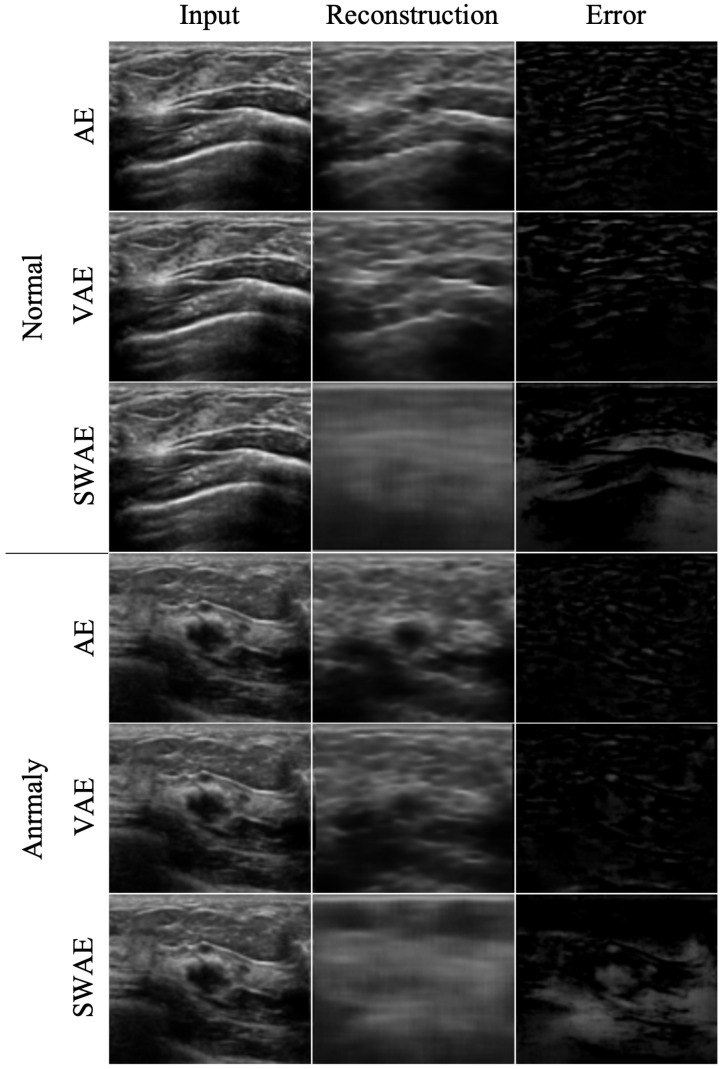
Reconstructed images by model.

The reconstruction performances of the models are presented in Table 4 by comparing the average RMSE of the verification process using normal ultrasound images and the average RMSE of abnormal ultrasound images. In the image reconstruction process by an AE, the smaller the RMSE value, the better the reconstruction performance. However, in a test process for abnormal ultrasonic images, a larger RMSE value indicates that the input image is not well-reconstructed. This means that the input image contains abnormal features that are difficult to reconstruct by the model. The pixel-wise differences between the input and reconstructed images would be suitable for identifying an anomalous region. In the comparison experiment for the three models, the RMSE value increases in the order of SWAE, VAE, and AE, and the anomalous region detection performance is found to be the best in the SWAE model. Examples of the image reconstruction results for each model are shown in Figure 9 below.

We confirmed that the AE model with the smallest RMSE value yielded restoration as the input. For the VAE model, although the normalization value was considered in learning, the results were similar to those of the AE model. This shows that it is difficult to find an anomalous region in an error image by restoring the anomalous region similar to the input as a result of the test by inputting an abnormal image. Conversely, the reconstructed images of the SWAE model, which showed the highest RMSE value in the evaluation process, did not restore abnormal features. The anomalous region could be verified in the different maps more accurately.

#### 4.2.2. Anomalous Region Detection

To evaluate the anomaly detection performance of the three models, we used three indicators, Dice, TPR, and FPR, as described in Section N. The results of detecting anomalous regions by the three models based on an arbitrary threshold of 0.2 are shown in Table 5.

**Figure 10:**
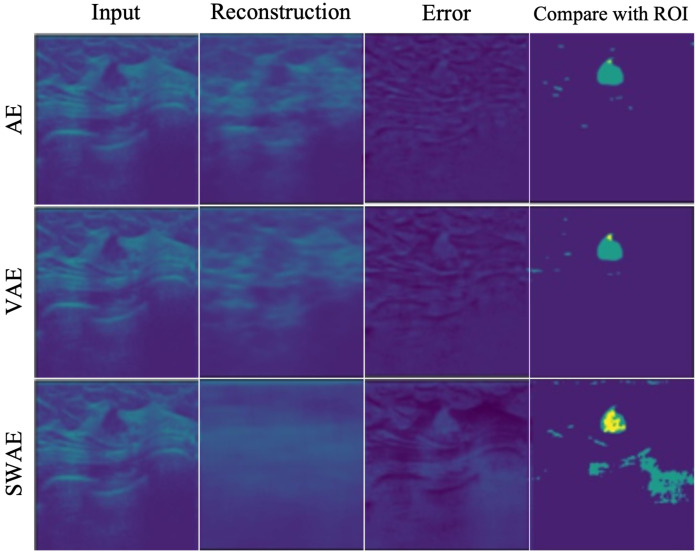
Reconstructed result images by models.

Similarity generally showed low values in the three models. However, they were the lowest in the AE model, and all indicator values showed the highest results in the SWAE model. The SWAE model showed relatively high sensitivity and good performance, but the FPR value was relatively low. Figure 10 shows each model’s anomalous region detection performance.

The AE model, which has the smallest similarity, sensitivity, and performance values, restored an input very similarly. It can be seen that there is almost no region indicating an abnormality in the case of binary division based on a specific threshold of 0.2. The VAE model restored the input image similar to the AE model, and both the error and binary-split images, and the indicator values, showed similar results to the AE model. The SWAE model shows the most significant result in all three indicator values. The anomalous region is most clearly detected and displayed in the error and binary-split images.

### 4.3. Analysis of Factor Influencing Anomalous Region Detection in Ultrasonography

#### 4.3.1. Threshold

**Figure 11:**
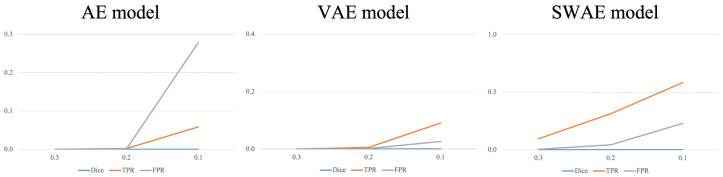
Changes in indicators according to the threshold for each model.

As a result of detecting anomalous regions of the models, the reconstruction-based approach is considerably affected by the threshold value. Figure 11 shows the change in indicators for each arbitrary threshold.

In all three models, the smaller the threshold, the larger the region, which is considered abnormal, indicating an increase in the TPR and FPR values. In the AE model, the FPR value increases significantly more than the TPR value because the FPR value, which considers typical abnormalities as normal, is larger than the TPR value, which considers abnormalities as abnormalities. It is difficult to say that the anomalous region was well-detected. The VAE and SWAE models show that the TPR value increases more than the FPR value as the threshold value decreases. In particular, for the SWAE model, the TPR value increases the most, indicating that the anomalous region was well-detected by considering the actual abnormality as abnormal. As shown in Figure 11, thresholds play an important role in anomalous region detection, thus, we did not use arbitrary thresholds. We applied the method using the validation data mentioned in Algorithm 1 of the Research Methodology to derive thresholds. The derived thresholds are shown in Table 6.

**Figure 12:**
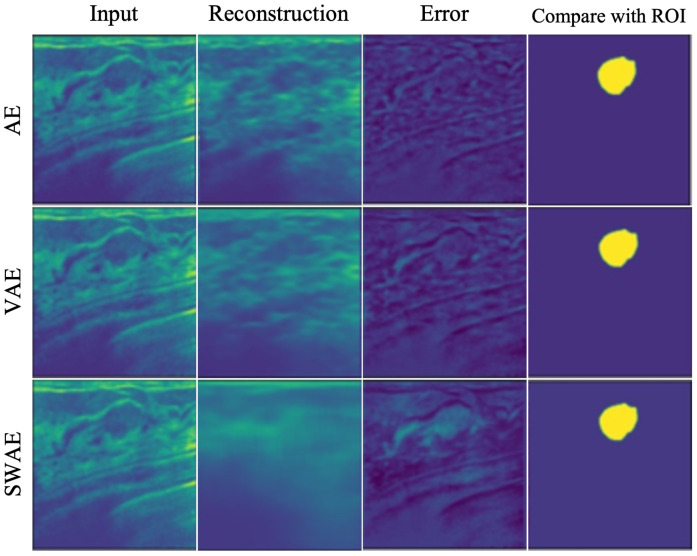
Anomalous region detection results with respect to threshold with applying Relu function.

The method applied in Table 6 uses the Relu function. The application method shows a relatively significant threshold value because the negative number is treated as 0 in the vector value of the error image. A significant threshold may occur in a region where the abnormality is treated as normal during the binary division of an error image. Figure 12 demonstrates the anomalous region detection results. Figure 12 shows that most results compared with the ROI are considered normal in the error image, resulting in the anomalous region not occurring and no overlapping area with the ROI occurring, which further indicates that it is difficult to detect the anomalous region.

When the average value of the verified data error image was used without applying the Relu function to calculate the threshold value for detecting the anomalous region of the breast ultrasonography, a threshold value, somewhat lower than that of applying the Relu function, was derived, indicating relatively good results for anomalous region detection. However, for small thresholds, the FPR value increases as the increase of FPs, indicating the limitation of anomalous detection.

#### 4.3.2. Size of Tumor

**Figure 13:**
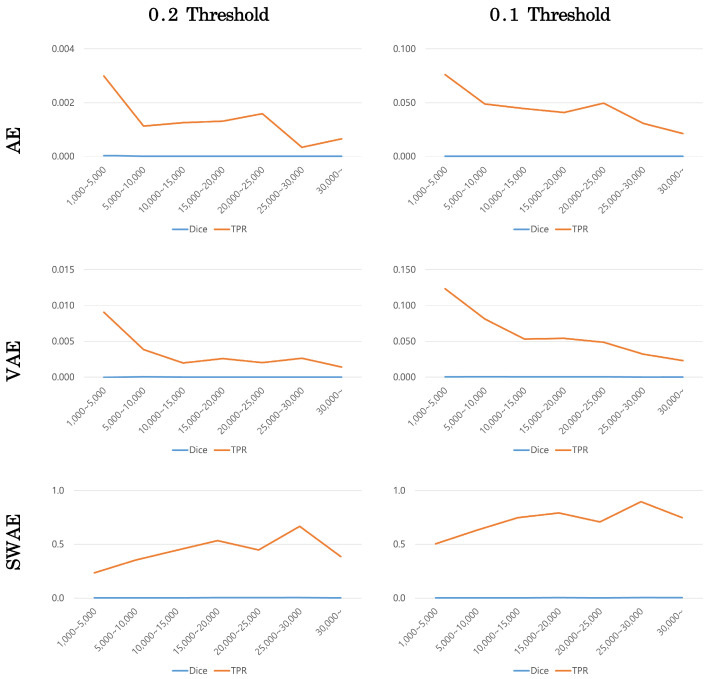
Changes in indicators according to tumor size by model.

The number of pixels in the ROI image representing the tumor was calculated to confirm the effect of tumor size on anomalous region detection. The tumor size was divided into ranges according to the number of pixels, and the averages of the Dice scores and TPR values in the corresponding range were calculated to compare the performance of each model. Figure 13 shows the change in indicators according to tumor size at a corresponding threshold for each model.

Dice scores were small in all models, making it difficult to compare, but TPR values showed similar patterns for each model. The error image is binary divided based on a specific threshold, hence, the TPR value can be calculated somewhat larger at a smaller threshold. However, the TPR value according to tumor size showed a similar pattern depending on the model’s threshold value. In the AE and VAE models, the TPR value decreased as the tumor size increased. Meanwhile, in the SWAE model, the TPR value increased as the tumor size increased to a specific range; in general, the larger the tumor size, the larger the TPR value.

## 5. Conclusions

In this study, we have used the reconstruction-based approach of unsupervised learning to confirm the effect of using deep learning-based technology to detect anomalies in breast ultrasound images. Three models–AE, VAE, and SWAE–were used to compare the results of anomalous region detection based on calculated specific threshold similarity (Dice), sensitivity (TPR), and FPR indicators. The performance results of restoring ultrasound images were good in the order of AE, VAE, and SWAE; however, abnormal images could not be restored in the anomalous region detection.

In addition, we confirmed that the SWAE model, which represents a more significant TPR value than the FPR value, exhibited relatively good performance in anomalous region detection. Meanwhile, the VAE model, which performed similar learning as the SWAE model by adding normalization values, failed to enforce the distribution of sample data, a characteristic of the model, resulting in similar results to the AE model.

The anomalous region detection technology applied in this study has a threshold-dependent limitation because based on a specific threshold, it determines whether an error image is abnormal by dividing it. This resulted in a higher TPR value with a decreasing threshold value. However, the FPR value that could detect non-tumor regions as tumors also increased and that was not a good result.

Changes in the Dice and TPR indicators according to the tumor size were confirmed to check the effect of tumor size on detecting anomalous regions. Although the indicator values might differ due to the difference in anomalous regions according to the threshold value, similar patterns were observed for each model. In the AE and VAE models, the larger the tumor size, the fewer the detected anomalous regions. This is observed as a result of a restoration similar to the anomalous region, resulting in a smaller region considered abnormal. Furthermore, because the reconstruction in the SWAE model was restored to map the anomalous region to normal, the overall anomalous region was detected. The larger the tumor size, the more overlapping parts occurred, and the higher the TPR value was.

In this study, we detected anomalous regions such as tumors and masses in ultrasound images and checked whether they could be visually presented. The results of anomalous region detection using the SWAE model showed the best performance in ultrasound images among the three AE-based models.

Further research is required to reduce learning through securing various samples, FPR values, and increasing TPR values to detect anomalous regions with improved performance on breast ultrasound images with high variance characteristics. Moreover, because the threshold setting considerably influences the anomalous region detection results, visual presentation of anomalous regions for ultrasound images will be possible if additional methods are applied to determine anomalies without a separate threshold setting.

## Figures and Tables

**Table 1:** Hyperparameter setting of the AE model.

Hyper Parameter	Value
Activation Function	LeakyReLU
Output Function	Sigmoid
Loss Function	L1 distance
Optimizer	Adam
Batch Size	16
Epochs	150
Learning Rate	0.0002

**Table 2:** Hyperparameter setting of the VAE model.

Hyper Parameter	Value
Activation Function	LeakyReLU
Output Function	Sigmoid
Loss Function	Reconstruction Error + KLD
Optimizer	Adam
Batch Size	16
Epochs	150
Learning Rate	0.0002

**Table 3:** Hyperparameter setting of the SWAE model.

Hyper Parameter	Value
Activation Function	LeakyReLU
Output Function	Sigmoid
Loss Function	Reconstruction Error + SWD
Optimizer	Adam
Batch Size	16
Epochs	150
Learning Rate	0.0002

**Table 4:** Reconstruction performances of models.

Model	Normal Ultrasound RMSE	Abnormal Ultrasound RMSE
AE	0.077	0.072
VAE	0.089	0.084
SWAE	0.139	0.139

**Table 5:** Indicators of anomalous region detection results of models.

Model	Similarity (Dice)	True Positive Rate (TPR)	False Positive Rate (FPR)
AE	0.000017	0.001995	0.001494
VAE	0.00005	0.005804	0.001616
SWAE	0.001252	0.312863	0.043162

**Table 6:** Comparison of thresholds by models.

Threshold	AE Model	VAE Model	SWAE Model
Applying Relu	0.52675	0.559735	0.497874

## Data Availability

Not applicable.
